# Early Functional Outcomes of Autologous Fibular Strut Graft Augmentation in Open Reduction and Internal Fixation (ORIF) of Proximal Humerus Fractures

**DOI:** 10.7759/cureus.103867

**Published:** 2026-02-18

**Authors:** Harjit Kanwar S Chawla, Amandeep S Bakshi, Davinder Singh, Jaspreet Singh, Mukul Sharma

**Affiliations:** 1 Orthopaedics, Government Medical College & Rajindra Hospital, Patiala, IND

**Keywords:** fibular strut allograft, fibular strut graft, functional outcome of philos plating in adults, osteoporotic fracture, philos, proximal humeral fracture

## Abstract

Background

Displaced proximal humerus fractures in elderly patients are associated with high rates of fixation failure due to poor bone quality and medial column comminution. Augmentation of locking plate fixation with an intramedullary fibular strut autograft has been proposed to enhance medial support and improve outcomes. This study evaluated early radiological and functional outcomes following open reduction and internal fixation (ORIF) using a proximal humerus internal locking system (PHILOS) plate augmented with a fibular strut autograft.

Objectives

The objectives of this study are to evaluate the early clinical and radiological outcomes of ORIF of displaced proximal humerus fractures using PHILOS plating augmented with an autologous intramedullary fibular strut graft, with emphasis on restoration of medial column support.

Methods

This prospective observational study included 50 patients with closed displaced proximal humerus fractures treated with ORIF using a PHILOS plate and intramedullary fibular strut autograft. Surgery was performed via a deltopectoral approach. Patients were followed at six weeks, three months, and six months postoperatively. Functional outcomes were assessed using the Constant-Murley score and Disabilities of the Arm, Shoulder and Hand (DASH) score. Radiological union, range of motion, and complications were recorded.

Results

The mean Constant-Murley score improved from 60.1 at six weeks to 71.9 at three months and 85.3 at six months (p < 0.05). Mean DASH scores decreased from 32.8 to 19.1 and 12.3 at corresponding intervals (p < 0.05). Early radiological union was achieved in all patients (100%). Shoulder range of motion at final follow-up approached near-normal values. One patient (2%) developed a superficial postoperative infection, which resolved with oral antibiotics. No major early complications were observed. No donor-site morbidity was noted.

Conclusion

Autologous fibular strut graft augmentation during ORIF of displaced proximal humerus fractures is associated with favorable early functional recovery and high early union rates. However, longer follow-up and comparative studies are required to assess long-term complications and durability.

## Introduction

Proximal humerus fractures account for approximately 5-6% of all fractures in adults and are increasingly encountered in elderly patients following low-energy falls. The healthcare burden associated with these fractures is significant and involves both the direct and indirect costs associated with osteoporosis and fragility fractures [1].

While minimally displaced fractures are typically managed non-operatively, the optimal treatment of displaced proximal humerus fractures remains controversial. A Cochrane review has highlighted the lack of high-quality randomized evidence favoring any single treatment modality [2]. Locking plate fixation has gained popularity due to improved angular stability; however, complications such as varus collapse, intra-articular screw penetration, non-union, and avascular necrosis remain common, particularly in osteoporotic bone [1,3,4].

Failure of the medial column support has been identified as a key factor contributing to fixation failure. Varus malalignment increases deforming forces exerted by the rotator cuff, leading to collapse and poor functional outcomes. Techniques aimed at restoring medial support include calcar screws, tuberosity tension band sutures, and controlled impaction. More recently, intramedullary fibular strut grafts have been introduced to provide structural medial buttress and enhance construct stability [5-7].

The present study was conducted to evaluate early functional outcomes, radiological union, maintenance of fracture alignment and short-term complications following ORIF of displaced proximal humerus fractures augmented with an intramedullary fibular strut autograft.

## Materials and methods

This prospective observational study was conducted at a tertiary care center over a one-year period from September 2024 to August 2025 following approval from the Institutional Ethics Committee. Written informed consent was obtained from all participants.

Patients of all age groups presenting with closed, displaced proximal humerus fractures were included in the study. Fractures were classified using the Neer classification system and displaced Neer two-part, three-part, and select four-part fractures meeting operative criteria were included. Patients with pathological fractures, open injuries, pregnancy, advanced shoulder osteoarthritis, malignancy, immunocompromised status, or those medically unfit for surgery were excluded. Demographic data, mechanism of injury, comorbidities, and fracture characteristics were recorded using a structured proforma.

**Figure 1:**
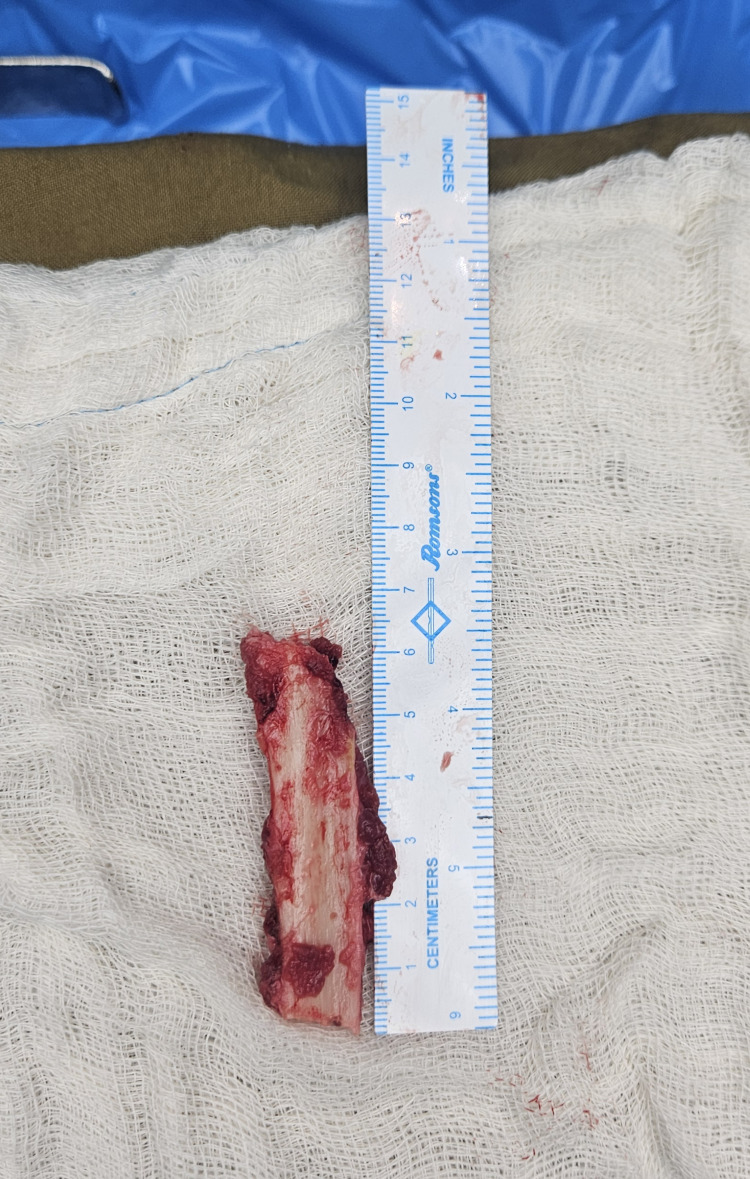
Intraoperative photograph showing the harvested autologous fibular strut graft obtained from the middle third of the fibula

**Figure 2:**
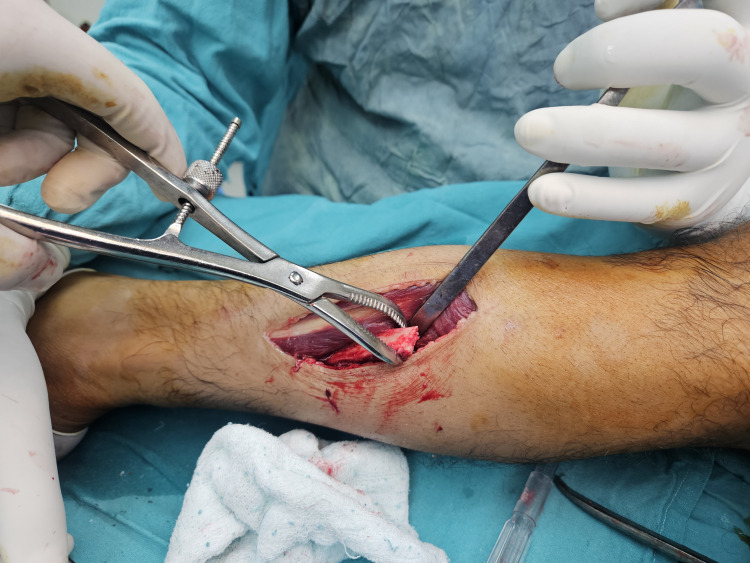
Intraoperative photograph demonstrating extraction of the autologous fibular strut graft through a separate lateral approach

Preoperative assessment included standard anteroposterior and lateral radiographs of the shoulder. All surgeries were performed under general or regional anesthesia using a standard deltopectoral approach, with the patient positioned in the beach-chair position. An autologous fibular strut graft was harvested from the ipsilateral leg through a separate lateral approach (Figure 1). A segment of fibula measuring approximately 6-8 cm was obtained from the middle third, preserving at least 6 cm proximally and distally to avoid ankle and knee instability (Figure 2). Following fracture reduction, the humeral canal was gently prepared. The harvested autologous fibular strut graft was contoured and press-fitted into the intramedullary canal, extending proximally to support the subchondral bone at the calcar region, thereby restoring medial column support.

**Figure 3:**
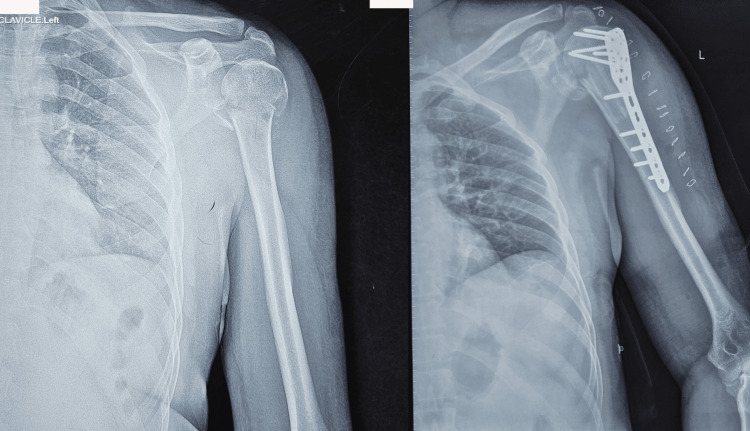
Preoperative radiograph of the shoulder demonstrating a displaced proximal humerus fracture and Immediate postoperative radiograph showing PHILOS plate fixation augmented with an autologous intramedullary fibular strut graft PHILOS: Proximal humerus internal locking system

The proximal humerus internal locking system (PHILOS) plate was applied in a standard fashion. Proximal locking screws were inserted into the humeral head, and where feasible, screws were passed through the fibular graft to enhance screw purchase and construct stability. Calcar screws were used whenever anatomy permitted (Figure 3). Care was taken to avoid excessive drilling of the graft to prevent graft fracture.

Postoperatively, the limb was supported in a sling. Passive range-of-motion exercises were initiated as per pain tolerance, followed by gradual progression to active-assisted and active movements. Patients were followed at six weeks, three months, and six months. Radiological outcomes were assessed using early fracture union, which was defined as bridging trabeculae across at least three cortices on orthogonal radiographs, combined with clinical outcomes such as absence of fracture-site tenderness and ability to perform pain-free functional shoulder movements. Early functional outcomes were assessed using the Constant-Murley score and the Disabilities of the Arm, Shoulder and Hand (DASH) score [5,8]. Statistical analysis was performed using IBM SPSS Statistics for Windows, Version 20 (Released 2015; IBM Corp., Armonk, New York, United States), with p < 0.05 considered statistically significant.

## Results

The mean age of patients was 62.9 years, with a male predominance (62%). Patients were stratified into <65 years and ≥65 years; both groups showed progressive functional improvement. The right shoulder was involved in 66% of cases (Table 1).

**Table 1:** Demographic characteristics of the study population

Variable	Number	Percentage
Mean age (years)	62.9	
Males	31	62
Females	19	38
Right side involved	33	66
Left side involved	17	34

Functional outcomes demonstrated progressive improvement over time. The Constant-Murley score increased significantly from six weeks to six months postoperatively (Table 2).

**Table 2:** Comparison of mean Constant-Murley scores at follow-up intervals

Follow-up	Mean constant score	p-value
Six weeks postoperative	60.1	0.001 (Significant)
Three months postoperative	71.9	
Six months postoperative	85.3	

DASH scores showed a corresponding and statistically significant decline, indicating an improved upper-limb function (Table 3).

**Table 3:** Comparison of mean DASH scores at follow-up intervals DASH: Disabilities of the Arm, Shoulder and Hand

Follow-up	Mean DASH score	p-value
Six weeks postoperative	32.8	0.000 (Significant)
Three months postoperative	19.1	
Six months postoperative	12.3	

Radiological union was achieved in all 50 patients (100%) with no cases of delayed union or non-union (Table 4).

**Table 4:** Radiological union outcomes

Union	Number	Percentage
Complete union	50	100
Non-union	0	0
Total	50	100

**Figure 4:**
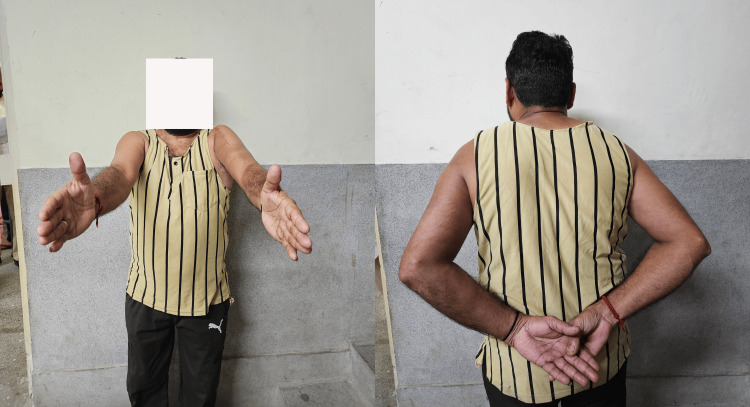
Clinical photograph demonstrating active range of motion of the operated shoulder at the six-month follow-up

At the final follow-up, shoulder range of motion on the affected side approached that of the contralateral limb (Table 5, Figure 4).

**Table 5:** Postoperative shoulder range of motion at the final follow-up

Variable	Mean
Active forward flexion (affected) (°)	149.1
Active forward flexion (contralateral) (°)	168.3
External rotation (affected) (°)	62.4
External rotation (contralateral) (°)	68.9

One patient developed a superficial postoperative infection, which was resolved with oral antibiotics. No neurovascular injuries, implant failures, or early signs of osteonecrosis were observed. No donor-site complications such as ankle instability, peroneal nerve injury, or persistent leg pain were observed during the follow-up period (Table 6).

**Table 6:** Postoperative complications and management

Complication	Number (%)	Management/Outcome
Superficial postoperative infection	1 (2)	Treated with oral antibiotics for two weeks; resolved completely

## Discussion

Management of displaced proximal humerus fractures remains challenging, particularly in elderly patients with osteoporotic bone and medial column comminution. Although locking plate fixation provides angular stability, high complication rates-including varus collapse, screw cutout, and intra-articular penetration-have been reported when medial column support is insufficient [9-11].

Biomechanically, loss of medial support leads to increased varus forces acting on the humeral head due to the lever arm of the rotator cuff, resulting in fixation failure. Restoration of medial column stability is therefore critical for maintaining reduction and improving functional outcomes. Intramedullary fibular strut allografts act as an internal buttress, resisting varus collapse and improving load sharing across the construct [12-19].

In the present study, fibular strut augmentation resulted in progressive improvement in shoulder function, with statistically significant gains in Constant-Murley scores and corresponding reductions in DASH scores over time. All fractures achieved radiological union, and no cases of implant failure or loss of reduction were observed. The relatively younger mean age of the cohort compared with typical osteoporotic populations may partially explain the high early union rates observed. This should be considered when interpreting the results. Reported union rates for fibular strut-augmented fixation range between 85% and 95% in published literature [16-18]. The high early union rate observed in the present study lies within the upper range of reported outcomes and should be interpreted in the context of short-term follow-up. No early clinical or radiographic signs suggestive of osteonecrosis were observed within the six-month follow-up period. The use of an autologous fibular strut graft provides biological advantages such as improved graft incorporation and elimination of disease transmission risk. Although donor-site morbidity is a concern, no such complications were observed in the present series during early follow-up. The low complication rate further supports the safety of this technique.

Our findings are consistent with previous clinical studies. Neviaser et al. demonstrated that endosteal fibular strut augmentation reduced complications associated with proximal humeral locking plates and improved functional outcomes [20]. Tan et al. reported early mobilization, maintained fracture alignment, and satisfactory functional recovery in elderly patients treated with fibular strut-augmented fixation [21]. A systematic review by Saltzman et al. concluded that fibular strut augmentation is associated with low complication rates and favorable mid-term outcomes, although higher-quality comparative studies are needed [22].

While the results of the present study are encouraging, limitations include the absence of a control group, relatively small sample size, inclusion of relatively younger patients and short follow-up duration. Long-term complications such as avascular necrosis or late implant failure could not be evaluated. Larger randomized studies with longer follow-up are necessary to establish definitive comparative efficacy. Nevertheless, the study provides prospective evidence supporting fibular strut augmentation as a useful adjunct in managing displaced proximal humerus fractures, particularly in patients with compromised bone quality.

## Conclusions

Autologous fibular strut graft augmentation during ORIF of displaced proximal humerus fractures provides stable fixation with excellent early union rates and progressive functional recovery. This technique appears to be a reliable adjunct for managing complex fractures associated with favorable early outcomes. However, larger comparative studies with longer follow-up are required.
